# Risk for Arterial Thromboembolic Events (ATEs) in Patients with Advanced Urinary Tract Cancer (aUTC) Treated with First-Line Chemotherapy: Single-Center, Observational Study

**DOI:** 10.3390/curroncol29090478

**Published:** 2022-08-24

**Authors:** Aristotelis Bamias, Kimon Tzannis, Roubini Zakopoulou, Minas Sakellakis, John Dimitriadis, Alkistis Papatheodoridi, Loukianos Rallidis, Panagiotis Halvatsiotis, Anna Tsiara, Maria Kaparelou, Efthymios Kostouros, Despina Barbarousi, Konstantinos Koutsoukos, Evangelos Fragiadis, Athanasios E. Dellis, Ioannis Anastasiou, Konstantinos Stravodimos, Alexandros Pinitas, Athanasios Papatsoris, Ioannis Adamakis, Ioannis Varkarakis, Charalampos Fragoulis, Stamatina Pagoni, Charis Matsouka, Andreas Skolarikos, Dionysios Mitropoulos, Konstantinos Doumas, Charalampos Deliveliotis, Constantinos Constantinides, Meletios-Athanasios Dimopoulos

**Affiliations:** 1 2nd Propaedeutic Dept of Internal Medicine, National & Kapodistrian University of Athens, ATTIKON University Hospital, Rimini st 1, 12642 Chaidari, Attiki, Greece; 2 Hellenic GU Cancer Group, Evrou st 89, 11527 Athens, Attiki, Greece; 3 Dept of Clinical Therapeutics, National & Kapodistrian University of Athens, ALEXNADRA Hospital, Vas. Sofias Ave 80, 11528 Athens, Attiki, Greece; 4 2nd Department of Cardiology, National & Kapodistrian University of Athens, ATTIKON University Hospital, Rimini st 1, 12642 Chaidari, Attiki, Greece; 5 Oncology Department, Athens General Hospital “G. Gennimatas”, Mesogeion 154, 11527 Athens, Attiki, Greece; 6 Haematology Division, Alexandra Hospital, Vasilissis Sofias 80, 11528 Athens, Attiki, Greece; 7 1st Dept of Urology, National & Kapodistrian University of Athens, LAIKON Hospital, Agiou Thoma st 17, 11527 Athens, Attiki, Greece; 8 2nd Dept of Surgery, Aretaieion Academic Hospital, National & Kapodistrian University of Athens, Vas. Sofias Ave 76, 11528 Athens, Attiki, Greece; 9 2nd Dept of Urology, National & Kapodistrian University of Athens, Sismanoglio General Hospital, Sismanoglou st 1, 15126 Athens, Attiki, Greece; 10 Department of Urology, Athens General Hospital “G. Gennimatas”, Mesogeion 154, 11527 Athens, Attiki, Greece

**Keywords:** arterial, thromboembolism, urinary tract, cancer, chemotherapy, observational

## Abstract

Arterial thromboembolism has been associated with cancer or its treatment. Unlike venous thromboembolism, the incidence and risk factors have not been extensively studied. Here, we investigated the incidence of arterial thromboembolic events (ATEs) in an institutional series of advanced urinary tract cancer (aUTC) treated with cytotoxic chemotherapy. The ATE definition included peripheral arterial embolism/thrombosis, ischemic stroke and coronary events. A total of 354 aUTC patients were analyzed. Most patients (95.2%) received platinum-based chemotherapy. A total of 12 patients (3.4%) suffered an ATE within a median time of 3.6 months from the start of chemotherapy. The most frequent ATE was ischemic stroke (n = 7). Two ATEs were fatal. The 6-month and 24-month incidence were 2.1% (95% confidence interval [CI]: 0.9–4.1) and 3.6% (95% CI: 1.9–6.2), respectively. Perioperative chemotherapy increased the risk for ATE by 5.55-fold. Tumors other than UTC and pure non-transitional cell carcinoma histology were also independent risk factors. No association with the type of chemotherapy was found. Overall, ATEs occur in 4.6% of aUTC patients treated with chemotherapy and represent a clinically relevant manifestation. Perioperative chemotherapy significantly increases the risk for ATE. The role of prophylaxis in high-risk groups should be prospectively studied.

## 1. Introduction

The association between cancer and clinical hypercoagulability is well-known. Cancer itself, through effects on hemostatic and fibrinolytic pathways, represents a major driver of thromboembolic risk, as evidenced by the wide variability in this risk by cancer type and stage [1,2,3,4]. Cancer-associated prothrombotic effects include the hematogenous release of cancer-derived microparticles that trigger the coagulation cascade, production of procoagulant factors such as factor X, release of mucins that activate platelets and endothelial cells through the binding of P-selectin, and stimulation of neutrophils to release decondensed chromatin that forms prothrombotic neutrophil extracellular traps [5,6,7]. This hypercoagulability state is associated with the development of both venous (VTEs) and arterial (ATEs) thromboembolic events. The former are more frequent and have been more extensively studied regarding both treatment as well as prevention [8,9,10,11,12,13,14]. In addition, Risk Assessment Models (RAMs) have been developed in order to identify cancer patients in high risk to develop VTE and thus could be candidates for prevention strategies [15,16]. On the contrary, data on ATEs are very limited. Furthermore, the development of these events in patients with cancer may be more complex than that of VTEs, where the most relevant predisposing factors are the malignancy itself or its treatment. Regarding ATEs, multiple other factors may also be relevant. Established risk factors for ATEs, such as smoking and hypertension, are more prevalent in specific cancer types, either as risk factors for the development of cancer, manifestations of the disease itself or as toxicity due to specific anti-cancer therapies. Effects of stress and frequent interruption of antithrombotics may also result in an increased risk for ATEs. Finally, taking into consideration that major ATEs, such as ischemic heart disease and stroke, are leading causes of death, it is plausible that the development of such events may impact on the prognosis of cancer patients [17].

Clinical series have suggested that ATEs may be common in patients with cancer [18,19,20,21]. A recent study, which used the Surveillance Epidemiology and End Results (SEER)-Medicare linked database, suggested that the risk of developing ischaemic heart disease and/or stroke differed among different types of cancer [1]. This has already been acknowledged in relation to the development of VTE incidence, which considerably differs among tumors of different origin [21]. Differences in the biology of the tumor, the agents used for systemic therapy and risk factors, which are common for the specific tumor type and for the development of arterial events, may result in distinctly different incidence of ATEs and implications for management and prevention. It is, therefore, useful to collect information on ATEs occurring in specific types of cancers rather than including multiple types of neoplastic diseases.

Advanced urinary tract cancer (aUTC) has been associated with frequent development of ATEs [22]. This is not surprising, taking into consideration the strong association of smoking with both aUTC and ATEs. Furthermore, platinum-based chemotherapy, which represents the standard systemic therapy in aUTC, is also thrombogenic [20]. The incidence and the risk factors associated with the development of ATEs in aUTC have been reported in three retrospective studies [1,10,23]. In two of them, all patients received platinum-based chemotherapy for locally advanced or metastatic disease and the incidence was 4% in both cases [10,23]. In the largest series of approximately 17,000 patients from the SEER database, a 7.1% cumulative incidence in 1 year from cancer diagnosis was reported. This series was considerably different than the previous two, since it predominantly included patients with non-metastatic disease; peripheral arterial disease (PAD) was not evaluated and information about the applied anti-cancer therapies was lacking. Currently, the accurate prediction of the probability of developing ATEs in patients with aUTC receiving cytotoxic chemotherapy and the identification of widely accepted risk factors represents an unmet medical need. For these reasons, we studied the incidence of ATEs in patients who received cytotoxic chemotherapy in our institution.

## 2. Methods

### 2.1. Study Design and Patient Population

This is an observational, cohort study. Patients with histologically confirmed UTC (bladder, urethra, renal pelvis or ureter) were selected from our institutional database according to the following criteria: advanced disease (clinical stages T4b [for bladder cancer] and/or N ≥ 2 and/or M1); transitional-cell, squamous or adenocarcinoma (pure or mixed) histology; treatment with at least one line of systemic chemotherapy for advanced disease. Clinical data were extracted from patient charts. The variables collected for this analysis are shown in Table 1. All patients gave their Institutional Review Board-approved written consent for the use of their medical data. 

The primary endpoint was objectively confirmed ATEs after the initiation of chemotherapy for advanced disease. The ATE definition included: peripheral arterial thrombosis/embolism, ischaemic stroke and coronary events (unstable angina or myocardial infarction [MI]). ATE had to be documented by at least one of the following methods: computerised tomography; magnetic resonance imaging, angiography. Major surgery was defined as any open or laparoscopic abdominal and/or pelvic surgery, including cystectomy. Disease was categorised as lymph-node/local (LNL) only (including only bladder or local relapse and/or lymph node metastases) and other (presence of non-lymph node metastases ± LNL disease).

### 2.2. Statistical Analysis

Cumulative incidence function (CIF) for ATE was calculated from the time of initiation of first-line chemotherapy and gives the proportion of patients at a specific time period who have an ATE, accounting for the fact that they may die before this. Thus, the CIF for ATE not only depends on the hazard of ATE but also on the hazard for death. CIF curves and competing events of death were presented in graphical form (Figure S1). The association of baseline and treatment-related factors with the development of ATE was assessed using competing-risk regressions [24]. Cause-specific hazard ratios from Cox models were estimated to be compared with Sub-distribution hazard ratios (SHRs) of Fine and Gray [24]. The latter was chosen as a better approach to acknowledge that patients may die before having an ATE.

Any covariates in the univariate analysis with a *p*-value ≤ 0.200 were evaluated in a multivariate, competing risk regression model. SHRs were calculated for each factor. The subgroup analyses were carried out using the same multivariate competing risks model. Schoenfeld residuals were calculated and plotted as a diagnostic measure of the model. Interactions and time-varying effects were also tested. Survival was computed by Kaplan–Meier curves and the impact of ATE on outcome was tested with the log-rank test. Subgroup analyses according to the type of ATE were also performed.

## 3. Results

Three hundred fifty-four patients who started first-line chemotherapy for aUTC from April 1995 to September 2015 at our institution were included in the analysis (Figure S2). Their pre-chemotherapy characteristics are shown in Table 1. Most patients received cisplatin-based (187, 53.1%) or carboplatin-based (150, 42.4%) first-line chemotherapy. The chemotherapy administered is depicted in detail in Table S1 [25,26,27,28,29,30,31,32,33,34,35,36,37]. The median follow up, following initiation of chemotherapy was 9.3 months (95% CI: 7.8–11). At the time of initiation of the first-line chemotherapy, 5.1% of patients were receiving anticoagulant therapy and 14.4% received antiplatelet agents. No patient had had a central venous catheter (CVC). Seventy-three patients (20.6%) received perioperative chemotherapy. Most patients (62%) did not receive any chemotherapy beyond the first-line.

### 3.1. Incidence of ATEs and Association with Clinical Characteristics

ATE occurrence according to patient demographics and clinical characteristics are shown in Table 1. Twelve patients (3.4%) suffered an ATE: peripheral arterial thrombosis/embolism: 2, ischemic stroke: 7, unstable angina: 1, non-ST elevation myocardial infarction (nSTEMI): 1, STEMI: 1. The median time from start of chemotherapy to the occurrence of the first ATE was 3.6 months (25th–75th percentile: 2.2–9). Four events (33.3%) occurred within the first 3 months, seven (58.3%) within the first 6 months and ten (83.3%) within the first year. The remaining two events (16.7%) occurred between 12 to 60 months after the initiation of first-line chemotherapy. Ten of the 12 patients received platinum-based first-line chemotherapy (cisplatin:5, carboplatin:5). One patient received vinflunine and the other gemcitabine/ifosfamide. The time of occurrence, management and outcomes of ATEs are depicted in Table S2. Two events (both ischaemic strokes) were fatal.

**Figure 1:**
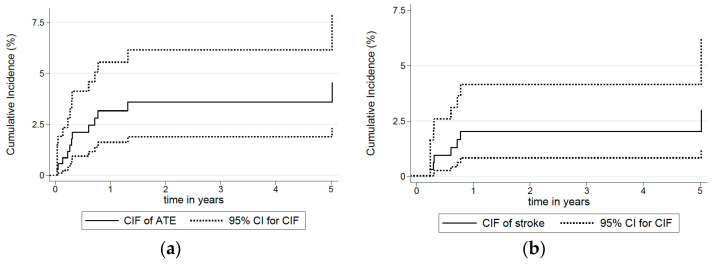
Cumulative incidence function (CIF) of all arterial thromboembolic events (ATEs) (**a**) and specifically for ischaemic strokes (**b**) over time from the initiation of first-line chemotherapy, with death as competing risk, for 354 patients treated for advanced urinary tract cancer. CI: confidence interval.

The cumulative 6-month and 24-month incidence of ATE were 2.1% (95% CI: 0.9–4.1) and 3.6% (95% CI: 1.9–6.2), respectively (Table 2). The absolute cumulative incidence over time from the initiation of first-line chemotherapy, with death as competing risk, is shown in Figure 1a. The CIF of ATEs at discrete time periods from the initiation of chemotherapy are shown in Table 2. Risk increased over time, up to 2 years after the initiation of chemotherapy.

The cumulative 6-month and 24-month incidence of ischaemic strokes was 1% (95% CI: 0.3–2.6) and 2% (95% CI: 0.8–4.2), respectively (Table 2). The absolute cumulative incidence over time from the initiation of first-line chemotherapy is shown in Figure 1b. Further subgroup analyses based on other types of ATEs were not performed due to the limited number of events in the other subgroups.

### 3.2. Association of ATEs with Type of Chemotherapy

Unadjusted cumulative ATE incidence functions based on the first-line chemotherapy regimen are shown in Table 2. Use of dose-dense regimen vs. conventional schedule chemotherapy, and cisplatin vs. carboplatin were not associated with increased risk of ATEs. Incidence of ATEs among gemcitabine-treated patients was almost 2-fold vs. those treated with non-gemcitabine-containing regimes (5.7% vs. 3.2%) but this difference was not statistically significant.

### 3.3. Uni- and Multivariate Analysis of ATE Risk

**Figure 2:**
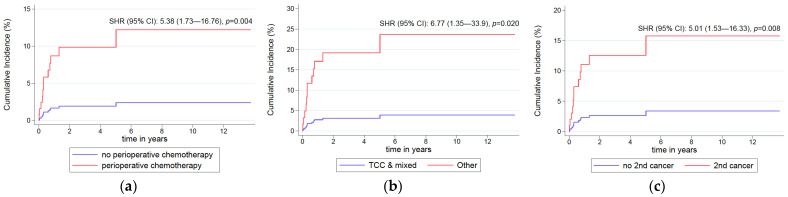
Competing-risks predicted cumulative incidence functions according to the presence of: (**a**) previous perioperative chemotherapy; (**b**) non-transitional-cell histology; (**c**) history of solid tumor other than urinary tract cancer (UTC). SHR: sub-distribution hazard ratio, CI: confidence interval.

In the univariate analysis, cumulative ATE incidence was significantly increased in patients with history of perioperative therapy, solid tumor other than UTC and those with histology other than transitional cell carcinoma (TCC) or mixed (Table 3). The respective cumulative ATE incidence functions at discrete time periods are shown in Table 2, while the absolute cumulative incidence over time from the initiation of the first-line chemotherapy, with death as competing risk, is depicted in Figure 2. Post first-line therapy was not associated with the development of ATE.

The three above variables were entered into a competing-risk cox regression model for multivariate analysis. Other factors, which could potentially be associated with increased thrombogenic activity, i.e., pre chemotherapy platelet count ≥ 3.50 × 10^11^/L, hemoglobin level < 10 g/dL or the use of red cell growth factors, pre-chemotherapy leukocyte count > 1.1 × 10^10^/L, BMI ≥ 35 kg/m^2^, anthracycline treatment, time since cancer diagnosis > 6 months, cardiovascular risk factors and comorbidities (composed by at least two of the following predictors: history of peripheral arterial embolism/thrombosis, coronary artery disease [CAD], hypertension, use of cholesterol-lowering drugs, diabetes) were also included in the model, although they were not associated with a *p* value ≤ 0.200 in the univariate analysis. The multivariate analysis (using the backward stepwise selection) confirmed the independent significance of all three factors identified in univariate analysis (Table 3). Interactions and time-varying effects of these variables were tested and none was found statistically significant. Regarding the clinical relevance of these factors, it is important to underline that only eleven patients had pure non-TCC histology, which accounted for only two of the total 12 ATEs. More importantly, both patients with pure non-TCC histology who developed ATE had received perioperative chemotherapy. Similar findings were observed for patients with tumors other than UTC: only one of the four patients suffering an ATE had not received perioperative chemotherapy.

## 4. Discussion

Cancer is frequently associated with an increase in thrombogenic potential. Although this might affect both venous and arterial circulation [21,38,39,40,41,42,43,44], ATEs have been largely disregarded in most studies of cancer-associated thrombosis (CAT). As a result, relative data for UTC are also very limited. Three large studies including mixed populations confirmed the significant increase in ATE risk in UTC compared to cancer-free controls [1,3,4]. In two smaller studies including only patients with aUTC who received platinum-based chemotherapy, the incidence was 4% in both cases [10,23]. In all studies, peripheral arterial thromboembolism was not included in the ATE incidence, while the three first studies included all stages, and the patients may not have received systemic therapy. Our study adds new information to these prior reports by including a homogenous population regarding cancer stage and therapy, while it is the first to include peripheral artery thromboembolism as an event in our calculations. We found a cumulative incidence of 4.6% close to that of the two previous smaller series [10,23]. The incidence reported by all three studies is lower than a 10.4% incidence at 2 years after the diagnosis of cancer, reported in a recent series from the SEER database [1]. This was true both for myocardial infarction as well as ischemic strokes. The reason for this discrepancy is not entirely clear. Several differences in patients’ selection exist between the SEER database analysis and the three other studies: patients from all stages and not only advanced disease were included in the first study, while details about the management (i.e., use of any therapy or not, type of therapy) were not available in the former. More importantly, the SEER population may represent a population in higher risk for cardiovascular disease: approximately 60% of patients had hypertension, atrial fibrillation or both, while the respective conditions in our population were 33% and 3%, respectively. The high risk of the SEER population is also suggested by the 8.7% risk for stroke or CAD of the controls at 2 years of follow up. These findings underline the importance of identifying risk factors for the development of ATEs among patients with specific types of cancer.

Ours is the first study to identify risk factors exclusively for the development of ATEs in aUTC, since previous studies studied composite (VTE and ATE) end points [10,23] or could not evaluate several of the parameters that we were able to [1]. We identified pure non-TCC histology, solid tumor other than UTC and history of perioperative chemotherapy as independent adverse factors for the development of ATE. The first has also been identified as a risk factor for venous thromboembolism but the mechanism underlying this association is obscure [45]. The association of the risk for ATE with the history of another tumor seems intuitive, since cancer is associated with increased risk for ATEs [1,2,3,4]. Nevertheless, the clinical relevance of these two factors is limited by the rarity of these conditions. Furthermore, all but one patient with at least one of these factors experiencing ATE also belonged to the group who received perioperative chemotherapy, suggesting that the third factor may be more relevant. A substantial 20% of our patients underwent neoadjuvant or adjuvant chemotherapy or both, and, according to contemporary trends, it is likely that this percentage is higher in current practice [46]. Approximately 10% of these patients suffered an ATE, accounting for seven of the twelve ATEs in the whole series. These findings might support the notion that specific populations who could be candidates for prevention strategies can be identified within the various types of cancer. The reason for the association we found is not clear. Patients who underwent perioperative chemotherapy did not have higher incidence of known factors predisposing for CAD, PAD or ischemic stroke. Thus, exposure to previous chemotherapy remains the most plausible factor to explain the association we found. Perioperative chemotherapy was platinum-based chemotherapy for all our patients. This type of therapy is thrombogenic [23]. It could, therefore, be speculated that double exposure to chemotherapy might increase the likelihood for ATE development.

Identifying risk factors predisposing for the development of ATEs is also important for the implementation of prophylaxis strategies. In contrast to VTEs, such research has not been extensive for ATEs. Incident cancer is not an established independent risk factor for arterial thromboembolism, and patients with cancer do not routinely receive therapies to prevent myocardial infarction and stroke [47,48,49]. Other factors also make this field more complicated, compared to VTEs: treatment of ATEs is not as homogenous as that of VTEs; equally medical treatment is not the only method of prevention, since surgery holds a significant role. Nevertheless, recent data add to the emerging consensus that arterial and venous thromboembolism are not quite as disparate as previously thought [50]. Common strategies for thromboprophylaxis could be designed, since anticoagulant therapy can also prevent ATEs. This notion is supported by the results of the CASSINI study, which demonstrated that rivaroxaban could reduce the composite incidence of venous and arterial thromboembolism in ambulatory patients with cancer [51]. Prophylaxis is particularly relevant in aUTC patients, since the high prevalence of cardiovascular risk factors, such as advanced age and smoking, expose them to high risk of cardiovascular events.

There are several limitations to our analysis. Despite being the largest institutional series of the specific population studied, the number of patients in our study is fairly small. Our study is limited by the inherent confounders and biases associated with any retrospective analysis. The validity of data regarding comorbidities and pre-existing medication was based on information from the patients and were not confirmed in all cases. Thus, some inaccuracies in reporting cannot be excluded. In this respect, the fact that all patients were treated in our institution represents an advantage, since we were able to review each file and limit inaccuracies to a certain extent. Specifically, we ensured that the diagnosis of ATEs had always been confirmed by standard imaging and was never based solely on patients’ information. Additionally, the treatment was fairly homogenous and the majority of patients had been treated with standard chemotherapy, while experimental targeted agents or immunotherapy were not included. Finally, our study cannot estimate the additive effect of chemotherapy to the risk of developing ATEs in patients with aUTC. This would require a non-chemotherapy cohort, which under the current guidelines, would not exist. In this respect, information provided by our study is in concert with current everyday practice and thus clinically relevant. 

In conclusion, our study offers important information on the incidence of and risk factors associated with the development of ATEs in aUTC. ATEs are less frequent than VTEs but may be a more complicated condition regarding both management and prevention. Since anti-coagulation may also prevent arterial thromboembolism, the field should consider abandoning the assessment of thromboprophylaxis exclusively in relation to VTEs and evaluate its role in ATE prevention among cancer patients. 

## Figures and Tables

**Table 1:** Baseline characteristics of 354 patients with advanced urothelial cancer who were included in the analyses.

**Characteristic**			**Median**	**Range**	
Age			67	32–88	
Weight			73.5	43–125	
BMI			26	15–51.1	
BSA			1.8	1.3–2.4	
Months after major surgery			6	1–180	
Cycles of chemotherapy			6	1–20	
**Characteristic**	**Total**		**ATE**		***p* Value**
	n	%	Yes (%)	No (%)	
Sex					0.230
Female	59	16.7	0 (0)	59 (17.3)	
Male	295	83.3	12(100)	283 (82.7)	
Diabetes					0.660
Yes	46	13	2 (16.7)	44 (12.9)	
No	308	87	10 (83.3)	298 (87.1)	
BMI >35					0.084
Yes	18	5.1	0 (0)	18 (5.3)	
No	334	94.4	11 (91.7)	323 (94.4)	
Missing	2	0.5	1 (8.3)	1 (0.3)	
BMI > 25					0.097
Yes	208	58.8	7 (58.3)	201 (58.8)	
No	144	40.7	4 (33.3)	140 (40.9)	
Missing	2	0.5	1 (8.3)	1 (0.3)	
Anti-platelet therapy					0.686
Yes	51	14.4	2 (16.7)	49 (14.3)	
No	303	85.6	10 (83.3)	293 (85.7)	
Anticoagulants					>0.999
Yes	18	5.1	0 (0)	18 (5.3)	
No	336	94.9	12 (100)	324 (94.7)	
Antihypertensives					0.773
Yes	137	38.7	4 (33.3)	133 (38.9)	
No	217	61.3	8 (66.7)	209 (61.1)	
Atrial fibrillation					>0.999
Yes	12	3.4	0 (0)	12 (3.5)	
No	342	96.6	12 (100)	330 (96.5)	
Cholesterol-lowering medication					>0.999
Yes	54	15.3	2 (16.7)	52 (15.2)	
No	300	84.8	10 (83.3)	290 (84.8)	
Coronary artery disease					>0.999
Yes	48	13.6	1 (8.3)	47 (13.7)	
No	306	86.4	11(91.7)	295 (86.3)	
Previous peripheral arterial embolism/thrombosis					0.336
Yes	13	3.7	1 (8.3)	12 (3.5)	
No	341	96.3	11 (91.7)	330 (96.5)	
Smoking history					0.952
Yes	174	49.2	6 (50)	168 (49.1)	
No	180	50.9	6 (50)	174 (50.9)	
Solid tumour other than UTC					0.022
Yes	35 ^1^	9.9	4 (33.3)	31 (9.1)	
No	319	90.1	8 (66.7)	311 (90.9)	
Previous VTE					>0.999
Yes	29	8.2	1 (8.3)	28 (8.2)	
No	325	91.8	11 (91.7)	314 (91.8)	
Haematologic malignancy					>0.999
Yes	3	0.9	0 (0)	3 (0.9)	
No	351	99.1	12 (100)	339 (99.1)	
Coagulation disorder					>0.999
Yes	2	0.6	0 (0)	2 (0.6)	
No	352	99.4	12 (100)	340 (99.4)	
Major Surgery					>0.999
Other major surgery (no cystectomy)	46	13	1 (8.3)	45 (13.2)	
Cystectomy	186	52.5	7 (58.4)	179 (52.3)	
No major surgery	122	34.5	4 (33.3)	118 (34.5)	
Time since UTC diagnosis					>0.999
≤6 months	12	3.4	0 (0)	12 (3.5)	
>6 months	342	96.6	12 (100)	330 (96.5)	
Histology					0.084
TCC	308	87	10 (83.3)	298 (87.1)	
Mixed	34	9.6	0 (0)	34 (10)	
non-TCC	11	3.1	2 (16.7)	9 (2.6)	
Missing	1	0.3	0 (0)	1 (0.3)	
Primary site					0.373
Bladder	298	84.2	12 (100)	286 (83.6)	
Bladder/Renal pelvis	2	0.5	0 (0)	2 (0.6)	
Renal pelvis	50	14.1	0 (0)	50 (14.6)	
Ureter	3	0.9	0 (0)	3 (0.9)	
Urethra	1	0.3	0 (0)	1 (0.3)	
Performance status					0.127
0	124	35	4 (33.3)	120 (35.1)	
1	132	37.3	8 (66.7)	124 (36.3)	
2	73	20.6	0 (0)	73 (21.4)	
3	25	7.1	0 (0)	25 (7.3)	
Number of disease sites					0.754
1	206	58.2	9 (75)	197 (57.6)	
2	107	30.2	3 (25)	104 (30.4)	
3	33	9.3	0 (0)	33 (9.7)	
4	8	2.3	0 (0)	8 (2.3)	
Location of disease					0.350
Pelvis	237	67	10 (83.3)	227 (66.4)	
Non-pelvis	117	33	2 (16.7)	115 (33.6)	
Type of Chemotherapy					
Cisplatin	188	53.1	5 (41.7)	183 (53.5)	0.139
Carboplatin	150	42.4	5 (41.7)	145 (42.4)	
Other	16	4.5	2 (616.7)	14 (4.1)	
Conventional	199	56.2	7 (58.3)	192 (56.2)	0.880
Dose-dense	155	43.8	5 (41.7)	150 (43.8)	
Gemcitabine	222	62.7	8 (66.7)	214 (62.6)	>0.999
Other	132	37.3	4 (33.3)	128 (37.4)	
Anthracycline	96	27.1	2 (16.7)	94 (27.5)	0.525
Non-anthracycline	258	72.9	10 (83.3)	248 (72.5)	
History of neoadjuvant/adjuvant chemotherapy					0.001
Yes	73	20.6	7 (58.3)	66 (19.3)	
No	281	79.4	5 (41.9)	276 (80.7)	
History of chemotherapy not for UTC					0.188
Yes	6	1.7	1(8.3)	5 (1.5)	
No	348	98.3	11 (91.7)	337 (98.5)	
History of radiation					>0.999
Yes	83	23.5	3 (25)	80 (23.4)	
No	271	76.5	9 (75)	262 (76.6)	
Radiation field					0.424
Pelvis	58	16.4	3 (25)	55 (16.1)	
Other + no radiation	296	83.6	9 (75)	287 (83.9)	
History of hormone therapy					>0.999
Yes	9	2.5	0 (0)	9 (2.6)	
No	345	97.5	12 (100)	333 (97.4)	
Hormone/anthracycline therapy					0.524
Yes	97	27.4	2 (16.7)	95 (27.8)	
No	257	72.6	10 (83.3)	247 (72.2)	
Pre-chemo PLTs > 350,000/μL					>0.999
Yes	113	31.9	4 (33.3)	109 (31.9)	
No	241	68.1	8 (66.7)	233 (68.1)	
Hgb < 10 g/dL or ESA					>0.999
Yes	34	9.6	1 (8.3)	33 (9.6)	
No	320	90.4	11 (91.7)	309 (90.4)	
Pre-chemo WBCs > 11,000/μL					0.704
Yes	66	18.6	1 (8.3)	65 (19)	
No	288	81.4	11 (91.7)	277 (81)	
ATE					
Yes	12	3.4			
No	342	96.6			
Type of ATE					
Peripheral arterial thrombosis/embolism	2				
Ischaemic stroke	7				
Unstable angina	1				
MI	2				
Subsequent lines of therapy					0.588
0	220	62.2	7 (58.3)	213 (62.3)	
1	82	23.1	2 (16.7)	80 (23.4)	
2–5	51	14.4	3 (25)	48 (14)	
missing	1	0.3	0 (0)	1 (0.3)	

BMI: body mass index; BSA: body surface area; ATE: arterial thromboembolic event; Fisher’s exact test; ^1:^ prostate: 17; lung: 4, breast: 3, head and neck: 3, colorectal: 2, parotid gland: 2, basal cell: 1, uterus: 1, thyroid: 1, seminoma: 1; UTC: urinary tract cancer; VTE: venous thromboembolic event; TCC: transitional-cell carcinoma; PLT: platelets; ESA: erythropoiesis stimulating agents; WBC: white blood cells; MI: myocardial infraction.

**Table 2:** Arterial thromboembolic (ATE) risk in 354 patients with advanced urinary tract cancer receiving first-line chemotherapy. Ιncidence function was calculated with death as a competing risk. The 95% confidence intervals are shown in parentheses.

	n (%)	Incidence Function (%)			
		3-Month	6-Month	12-Month	24-Month
Total ATE cases	12 (100)	1.2 (0.4–2.8)	2.1 (0.9–4.1)	3.2 (1.6–5.6)	3.6 (1.9–6.2)
Ischaemic stroke	7 (58.3)	0.3 (0.3–1.6)	1 (0.3–2.6)	2 (0.8–4.2)	2 (0.8–4.2)
Cisplatin					
Yes	188 (53.1)	1.1 (0.2–3.6)	2.3 (0.8–5.4)	2.9 (0.8–5.4)	2.9 (1.1–6.3)
No	166 (46.9)	1.3 (0.3–4.1)	1.9 (0.5–5.1)	3.5 (1.3–7.4)	4.4 (1.8–8.8)
Dose dense chemotherapy					
Yes	155 (43.8)	1.3 (0.3–4.3)	2.8 (0.9–6.6)	3.6 (1.3–7.7)	3.6 (1.3–7.7)
No	199 (56.2)	1 (0.2–3.4)	1.6 (0.4–4.2)	2.8 (1.1–6.1)	3.6 (1.5–7.2)
Gemcitabine					
Yes	222 (62.7)	1.4 (0.4–3.8)	2.5 (0.9–5.3)	3.1 (1.3–6.3)	3.9 (1.7–7.5)
No	132 (37.3)	0.8 (0.1–3.8)	1.6 (0.3–5.1)	3.2 (1.1–7.5)	3.2 (1.1–7.5)
Histology					
TCC + mixed	342 (96.9)	0.6 (0.1–2.1)	1.6 (0.6–3.5)	2.7 (1.3–5)	3.1 (1.5–5.7)
Other	11 (3.1)	18.2 (2.9–44.2)	18.2 (2.9–44.2)	18.2 (2.9–44.2)	18.2 (2.9–44.2)
Solid tumour other than UTC					
No	319 (90.1)	1.3 (0.4–3.1)	1.7 (0.6–3.6)	2.4 (1.1–4.7)	2.9 (1.3–5.4)
Yes	35 (9.9)	-	6.5 (1.2–18.7)	11 (2.7–25.9)	11 (2.7–25.9)
History of adjuvant/neoadjuvant					
None	281 (79.4)	0.8 (0.2–2.5)	1.2 (0.3–3.1)	1.6 (0.5–3.8)	2.1 (0.8–4.7)
One at least	73 (20.6)	2.8 (0.5–8.6)	5.7 (1.8–12.8)	9 (3.7–17.4)	9 (3.7–17.4)

**Table 3:** Univariate, multivariate, cox and competing risk regression analysis of arterial thromboembolism based on clinical characteristics. Variables with *p* < 0.200 are presented.

	Univariate							Multivariate		
	Cox Regression Analysis				Competing Risks Analysis			Competing Risks Analysis		
Factor	n	HR	95% CI	*p*	SHR	95% CI	*p*	SHR	95% CI	*p*
Solid tumour other than UTC				0.010			0.008			0.028
No	319	Ref			Ref			Ref		
Yes	35	4.88	1.46–16.3		5.01	1.53–16.33		3.71	1.15–11.97	
Histology				0.003			0.020			0.028
TCC + mixed	342	Ref			Ref			Ref		
Other	11	10.15	2.16–47.68		6.77	1.35–33.9		7.79	1.25–48.43	
History of adjuvant/neoadjuvant				0.005			0.004			0.010
None	281	Ref			Ref			Ref		
One at least	73	5.17	1.63–16.41		5.38	1.73–16.76		5.55	1.51–20.48	

UTC: urinary tract cancer; HR: hazard ratio; SHR: sub-distribution hazard ratio; CI: confidence interval.

## Data Availability

The data presented in this study are available upon reasonable request from the corresponding author. The data are not publicly available.

## Supplementary Materials

The following supporting information can be downloaded at: https://www.mdpi.com/article/10.3390/curroncol29090478/s1, Figure S1: Stacked Cumulative Incidence functions (CIF) of arterial thromboembolic events (ATE) and death over time; Figure S2: Study flow; Table S1: Detailed description of 1st-line chemotherapy administered to the 354 patients included in the analysis; Table S2: Management and outcomes of the 12 arterial thromboembolic events (ATEs), which occurred among 354 patients with advanced urothelialcancer, treated with platinum-based chemotherapy.

## Abbreviations

VTEs	Venous thromboembolic events
ATEs	Arterial thromboembolic events
RAMs	Risk Assessment Models
SEER	Surveillance Epidemiology and End Results
aUTC	Advanced urinary tract cancer
PAD	Peripheral arterial disease
LNL	Lymph-node/local
CIF	Cumulative incidence function
SHR	Sub-distribution hazard ratio
CVC	Central venous catheter
nSTEMI	Non-ST elevation myocardial infarction
STEMI	ST elevation myocardial infarction
TCC	Transitional cell carcinoma
CAD	Coronary artery disease
CAT	Cancer-associated thrombosis
