# Supplementary material for: Risk for Arterial Thromboembolic Events (ATEs) in Patients with Advanced Urinary Tract Cancer (aUTC) Treated with First-Line Chemotherapy: Single-Center, Observational Study
Source: Curr Oncol. 2022 Aug 24;29(9):6077–90. doi: 10.3390/curroncol29090478 (PMC9498031; doi:10.3390/curroncol29090478)
Supplement: Supplementary file 1 [file curroncol-29-00478-s001.zip › curroncol-1845019-supplementary.pdf]

Supplementary Material

Risk for Arterial Thromboembolic Events (ATEs) in Patients with Advanced Urinary Tract Cancer (aUTC) Treated with First-Line Chemotherapy: Single-Center, Observational Study

Aristotelis Bamias [1,2,*], Kimon Tzannis [1,2], Roubini Zakopoulou [3], Minas Sakellakis [2], John Dimitriadis [3], Alkistis Papatheodoridi [3], Loukianos Rallidis [4], Panagiotis Halvatsiotis [1], Anna Tsiara [3], Maria Kaparelou [3], Efthymios Kostouros [5], Despina Barbarousi [6], Konstantinos Koutsoukos [3], Evangelos. Fragiadis [7], Athanasios E. Dellis [8], Ioannis Anastasiou [7], Konstantinos Stravodimos [7], Alexandros Pinitas [9], Athanasios Papatsoris [9], Ioannis Adamakis [7], Ioannis Varkarakis [9], Charalampos Fragoulis [10], Stamatina Pagoni [5], Charis Matsouka [5], Andreas Skolarikos [9], Dionysios Mitropoulos [7], Konstantinos Doumas [10], Charalampos Deliveliotis [9], Constantinos Constantinides [7] and Meletios-Athanasios Dimopoulos [3]

Fig S1

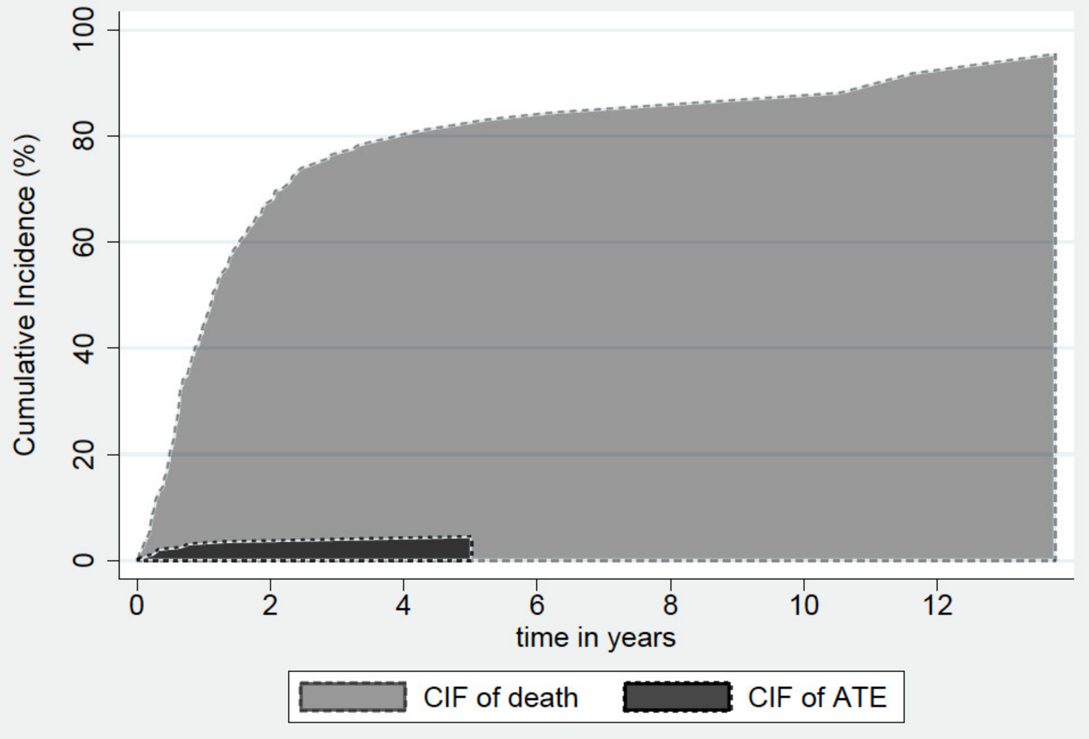

Figure S1. Stacked Cumulative Incidence functions (CIF) of arterial thromboembolic events (ATE) and death over time.

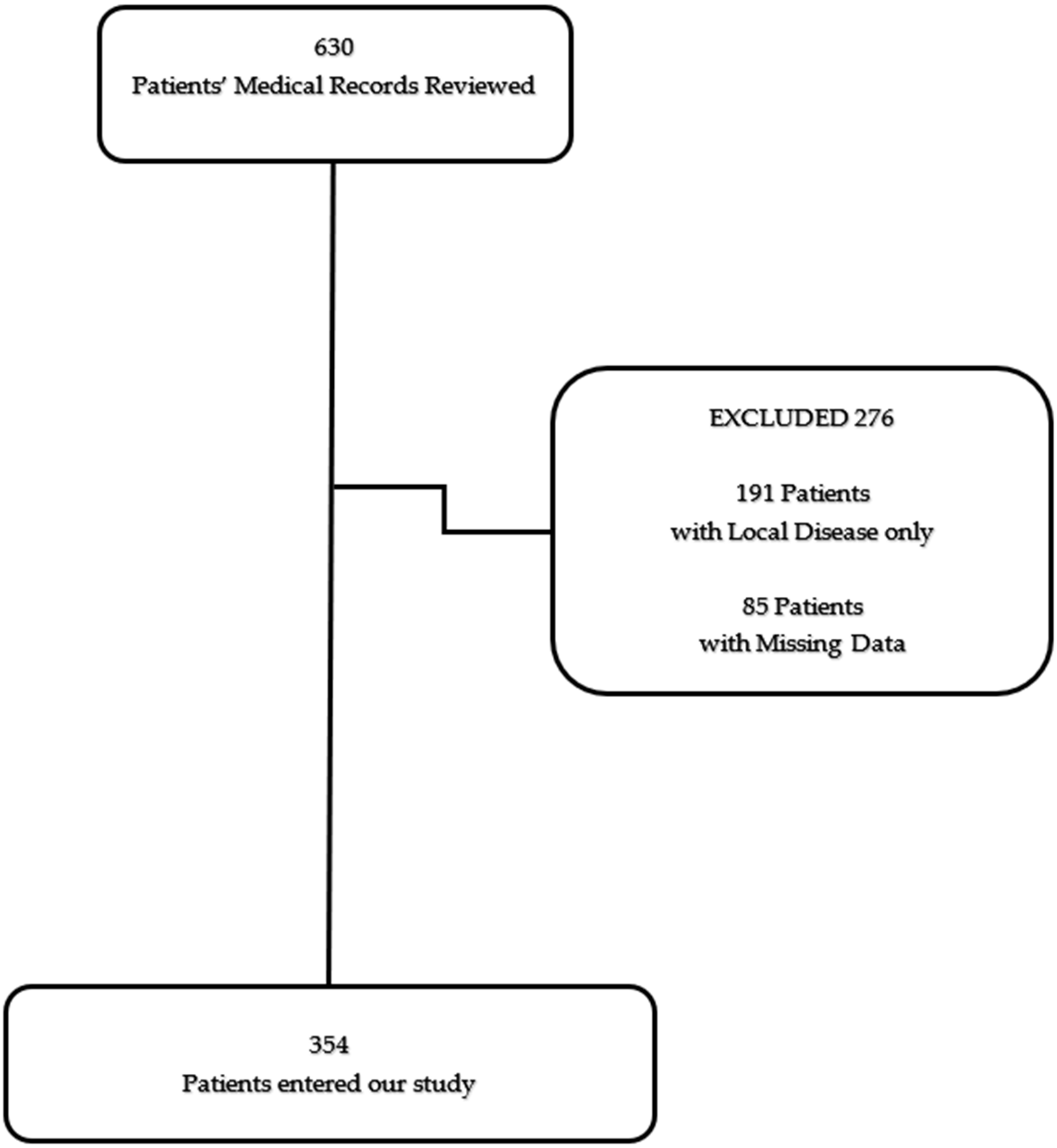

Figure S2. Study flow.

Table S1. Detailed description of 1st-line chemotherapy administered to the 354 patients included in the analysis.

**DdGC**[25]	**70**
**ddMVAC**[25]	84
**Docetaxel-Gemcitabine**[26]	5
**Docetaxel**[27,28]	2
**Paclitaxel-Gemcitabine**[29]	1
**Gemcitabine**[27,28]	1
**Gemcitabine-Ifosfamide**[30]	2
**Gem-Carbo**[31]	136
**MCaVi**[32]	6
**CMV-Ifosfamide**[33]	1
**MVAC**[34]	12
**GC**[34]	6
**Paclitaxel-Carbo**[35]	7
**Carbo**[27,28]	1
**Vinflunine**[36]	2
**Vinorelbine**[27,28]	4
**Docetaxel-Cisplatin**[37]	14

DdGC (dose dense Gemcitabine, Cisplatin), ddMVAC (dose dense Methotrexate, Vinblastine, Doxorubicin, Cisplatin), Gem- Carbo (Gemcitabine- Carboplatin), MCaVi (Methotrexate, Carboplatin, Vinblastine), CMV- Ifosfamide (Cisplatin, Methotrexate, Vinblastine – Ifosfamide), MVAC (Methotrexate, Vinblastine, Doxorubicin, Cisplatin), GC (Gemcitabine- Cisplatin), Paclitaxel- Carbo (Paclitaxel, Carboplatin), Carbo (Carboplatin).

Table S2. Management and outcomes of the 12 arterial thromboembolic events (ATEs), which occurred among 354 patients with advanced urothelial cancer, treated with platinum-based chemotherapy.

Type of ATE	n	Months from Chemotherapy Initiation Median (95% CI)	Management of ATE	n	Outcome of ATE	n
Ischemic stroke	7	7.3 (2.7–9.3)	Acenocoumarol	1	Death	1
			Antiplatelet	2	Resolution/Improvement	2
			Endarterectomy	1	Resolution/Improvement	1
			Unknown	3	Death	1
					Resolution/Improvement	1
					unknown	1
Coronary artery event	3	0.6 (0.3–NR)	Conservative	3	Resolution/Improvement	1
					unknown	2
Arterial embolism	2	1.6 (1.6–NR)	Conservative	2	Improvement	2

CI: confidence interval; NR: not reached.
